# Draft Genome Sequence of *Pseudomonas putida* JLR11, a Facultative Anaerobic 2,4,6-Trinitrotoluene Biotransforming Bacterium

**DOI:** 10.1128/genomeA.00904-15

**Published:** 2015-09-03

**Authors:** Javier Pascual, Zulema Udaondo, Lazaro Molina, Ana Segura, Abraham Esteve-Núñez, Antonio Caballero, Estrella Duque, Juan Luis Ramos, Pieter van Dillewijn

**Affiliations:** a Department of Environmental Protection, Estación Experimental del Zaidín, Consejo Superior de Investigaciones Científicas, Granada, Spain; b Abengoa Research, Department of Biotechnology, Sevilla, Spain; c Department of Chemical Engineering, University of Alcalá, Alcalá de Henares, Madrid, Spain

## Abstract

We report the draft genome sequence of *Pseudomonas putida* JLR11, a facultative anaerobic bacterium that has been studied in detail for its capacity to use the explosive 2,4,6-trinitrotoluene (TNT) as a nitrogen source. The sequence confirms the mechanisms used by this versatile strain to reduce and assimilate nitrogen from TNT.

## GENOME ANNOUNCEMENT

*Pseudomonas putida* JLR11 was originally isolated from a water treatment plant in Granada (Spain) as a bacterial strain capable of growth with 2,4,6-trinitrotoluene (TNT) as a sole nitrogen source under anaerobic conditions (1), using the nitrite released during the reduction of the TNT as a final electron acceptor for respiratory chains (2). Further detailed studies have revealed how this bacterium may obtain and assimilate nitrogen from this recalcitrant and toxic nitroaromatic compound (3–6). Additionally, its use for the bioremediation of TNT-contaminated soils has also been evaluated (7).

Genomic DNA was extracted from *P. putida* JLR11 using the Wizard Genomic DNA purification kit. The DNA was then sequenced by a paired-end Illumina MiSeq (basic biology service of the University of Granada, Spain) and Roche/454 pyrosequencing method on the Genome Sequencer FLX system with Titanium chemistry (Macrogen, Inc., South Korea). Quality control (FastQC version 0.52), trimming, and assembly were carried out under the Orione framework (8). After filtering and trimming the reads (9), average coverages of 17× and 26× were obtained for the Illumina MiSeq and Roche/454 pyrosequencing libraries, respectively. Illumina MiSeq reads were assembled with Velvet Optimizer vlsci version 1.0.0, and the Roche/454 pyrosequencing reads were assembled with the MIRA v4.0 *de novo* assembler version 0.0.4 (10, 11). Finally, contigs generated by both strategies were integrated with CISA Contigs Integrator version 1.0.1. The contigs were aligned and ordered against *P. putida* KT2440 (PRJNA57843) using Mauve version 2.4.0 (12). Automated genome annotation was carried out using the NCBI Prokaryotic Genomes Annotation Pipeline (PGAP) (http://www.ncbi.nlm.nih.gov/genome/annotation_prok) (13), as well as the Integrated Microbial Genomes Expert Review (IMG-ER) platform (14).

The assembled draft genome consists of 39 scaffolds (size, >1.077 bp) with a total size of 6,100,369 bp, an *N*_50_ contig length of 529,984 nucleotides, and a mean G+C content of 61.58%. A total of 5,368 coding DNA sequences, 113 pseudogenes, 59 tRNAs, 7 rRNAs (5S, 16S and 23S), and 1 noncoding RNA (ncRNA) were annotated. Three clustered regularly interspaced short palindromic repeat arrays were predicted. Average nucleotide identity analysis revealed that the draft genome is 99% identical to that of *P. putida* KT2440.

Strain JLR11, like other *Pseudomonas putida* strains, utilizes the Entner-Doudoroff pathway for the metabolism of hexoses. It codifies for a number of nitroreductases involved in the reduction of the TNT, most notably PnrA and XenB. The first efficiently reduces the nitro groups of the TNT to 4-ADNT (4), whereas the second, an old yellow enzyme, has the additional ability to reduce the aromatic ring of TNT to produce Meisenheimer complexes leading to the release of nitrite (6). This nitrite is reduced to ammonia with the assimilatory nitrite reductase NasB (3), which possibly may allow for anaerobic respiration when oxygen is depleted. The ammonia generated from the nitrite reduction is assimilated to glutamine by glutamine synthetase (*glnA*) and further to glutamate with glutamate synthase (NADPH/NADH) (*gltB*-*gltD*) or alternatively with glutamate dehydrogenase (NADP+) (*gdhA*) (3).

### Nucleotide sequence accession number.

The draft-genome sequence of *Pseudomonas putida* JLR11 was deposited at DDBJ/EMBL/GenBank under the accession number LDJF00000000. The version described in this paper is the first version.
